# Therapeutic Effect of Vagus Nerve Stimulation on Depressive-Like Behavior, Hyperglycemia and Insulin Receptor Expression in Zucker Fatty Rats

**DOI:** 10.1371/journal.pone.0112066

**Published:** 2014-11-03

**Authors:** Shaoyuan Li, Xu Zhai, Peijing Rong, Michael F. McCabe, Xing Wang, Jingjun Zhao, Hui Ben, Shuxing Wang

**Affiliations:** 1 Department of Physiology, Institute of Acupuncture and Moxibustion, China Academy of Chinese Medical Sciences, Beijing, China; 2 Department of Anatomy, Xinxiang Medical University, Xinxiang, Henan, China; 3 Department of Anesthesia, Critical Care, and Pain Medicine, Massachusetts General Hospital, Harvard Medical School, Boston, MA, United States of America; 4 Primate Research Center, Guangdong Entomological Institute, Guangzhou, Guangdong, China; 5 Guangdong Landau Biotechnology Co. Ltd, Guangzhou, Guangdong, China; University of Regensburg, Germany

## Abstract

Depression and type 2 diabetes (T2D) are common comorbid diseases and highly prevalent in the clinical setting with an unclarified mechanism. Zucker diabetic fatty (ZDF, fa/fa) rats natively develop T2D with hyperglycemia and hyperinsulinemia. Here we studied whether ZDF rats also innately develop depression, what a correlation is between depression and T2D, whether insulin receptor (IR) expression is involved in, and whether transcutaneous auricular vagus nerve stimulation (taVNS) would be beneficial in amelioration of the comorbidity. Six week old male ZDF and Zucker lean (ZL, fa/+) littermates were randomly divided into naïve (ZDF, n = 6; ZL, n = 7) and taVNS (ZDF-taVNS, n = 8; ZL-taVNS, n = 6) groups. Once daily 30 min-taVNS sessions were administrated under anesthesia for 34 consecutive days in taVNS groups. Blood glucose levels were tested weekly, and plasma glycosylated hemoglobin (HbAlc) level and immobility time in forced swimming test were determined on day 35 in all groups. The expression of insulin receptor (IR) in various tissues was also detected by immunostaining and Western blot. We found that naïve ZDF rats developed hyperglycemia steadily. These ZDF rats showed a strong positive correlation between longer immobility time and higher plasma HbAlC level. Long term taVNS treatment simultaneously prevented the development of depression-like behavior and progression of hyperglycemia in ZDF rats. The expression of IR in various tissues of naïve ZDF rats is lower than in naïve ZL and long-term taVNS treated ZDF rats. Collectively, our results indicate that in ZDF rats, i) depression and T2D develop simultaneously, ii) immobility time and HbAlc concentrations are highly and positively correlated, iii) a low expression of IR may be involved in the comorbidity of depression and T2D, and iv) taVNS is antidiabetic and antidepressive possibly through IR expression upregulation.

## Introduction

Type 2 diabetes (T2D) is a metabolic disorder characterized by high blood sugar level in the context of insulin resistance, in which body cells have lost the ability to respond adequately to relatively normal levels of insulin [1]. Rates of T2D have been increasing markedly since 1960 in parallel with obesity. To make things worse, ongoing epidemiological studies estimate that greater than 60% of adult US population may be categorized as either overweight or obese, which is a major risk factor of T2D [2], [3]. Furthermore, there is a growing appreciation that the complications of obesity extend to the central nervous system (CNS) and may result in increased risk for neurological comorbidities such as depressive illness. Depression is a state of low mood that affects thoughts, behavior, feelings and sense of well-being. Patients with depression may lose interest of life and contemplate, attempt, or commit suicide [4].

During the past two decades, studies found an increased prevalence of depression in the diabetic population, and vice versa [4], [5]. Despite the high prevalence of and common comorbidity between depression and T2D [6], [7], the mechanism behind the phenomena is unclear. The increasing burden of T2D and major depressive disorder makes the search for an extended understanding of etiology and for the development of additional treatments highly significant.

As a kind of complementary and alternative medicine, acupuncture is generally accepted as a beneficial, well-tolerated, and safe monotherapy for depression in animal models, clinical patients, and eldercare facility residents [8]–[11]. Specifically, the taVNS, which stimulates the afferent auricular branches of vagus nerve that project to solitary nucleus [12]. Neurons in solitary nucleus further project, mono- or multi-synaptically, to the limbic and the autonomic nervous system structures, including the pineal gland, ventral tegmental area, the hypothalamus, amygdala, anterior cingulate cortex, nucleus accumbens, and the lateral prefrontal cortex [12]. Fibers from the solitary nucleus also project to the locus ceruleus and dorsal raphe nucleus, respectively major nuclei related to noradrenergic and serotonergic innervations of the entire brain cortex. It is clear that the serotonergic, dopaminergic, and noradrenergic systems are commonly involved in the pathophysiology of depression and in the neuromechanisms of action of antidepressants [12]. Acupuncture has also been shown to decrease blood glucose level in T2D [13] and is beneficial in the treatment of obesity [14], [15], which is a primary cause of T2D in genetically predisposed people [3].

Based on the existence of overt depression in Zucker diabetic fatty (ZDF, fa/fa) rats [12] as well as the increasingly prevalent and high comorbidity of obesity and depression [16], in this study we used ZDF rats as a diabetes and depression rodent model to explore the correlation of the illnesses, the antidiabetic and antidepressive effectiveness of taVNS, and focused on a possible involvement of IR expression in the mechanism of the comorbidity between T2D and depression.

## Materials and Methods

### Animal model

Male ZDF rats (n = 14) and ZL littermates (n = 13) were purchased from Vital River Laboratories International Inc. (Beijing, China). Littermates from the same or foster mother were housed in one large cage with water and normal food pellets available ad libitum except during taVNS session. Animal room was maintained at 22±1°C and artificially illuminated from 7:00 A.M. to 7:00 P.M. There was no previous handling of the rats. Rats entered the experimental procedure at 6 weeks of age and were separated into ZDF and ZL groups according to the genotype, the appearance, and the first tested glucose concentration. The initial body weight was 175±15 grams for ZDF and 130±10 for ZL rats. The rats were raised in groups of three to four. The cages and beddings were changed every other day. The person in charge of the animal's welfare was also the same person who carried out the experiments. The experimental protocol was approved by Institutional Animal Care and Use Committee in China Academy of Chinese Medical Sciences.

### Blood glucose concentration test

By using Ascensia Breeze Blood Glucose Monitoring System (Newbury, Berks, UK), the progression of T2D was determined by weekly tested non-fasted blood glucose concentration from week 0 (baseline) to week 5. In an electronic stimulation experiment room, a rat was tenderly put into the plexiglass box linked to the inhalation anesthesia system. Under 2% isoflurane anesthesia, glucose concentration was tested from tail tip blood immediately before taVNS session. The test range was 0.6–33.3 mmol/L. Any concentration over the testing limit was recorded as 33.3 mmol/L for statistical purposes.

### Administration of taVNS

All the time points recorded in this study are in accordance with the taVNS occurrences, i.e. the beginning of taVNS is day 1. For taVNS, under 2% isoflurane inhalation anesthesia, two opposite magnetic electrodes (+/−) were placed over the right side auricular concha region, inside and outside respectively so that electronic current can conduct through the tissue, including the vagus nerve fibers. Saline was applied between an electrode and the skin to improve electronic conduction. A 30 min taVNS procedure at a frequency of 2/15 Hz (2 and 15 Hz, switched every second) and an intensity of 2 mA was administered once daily via an electrical stimulator (HANS-100, Nanjing, China). The parameters had been proven effective previously [17]. The procedures were performed in the afternoon after blood glucose test and blood sample collection at designed time points, for 34 consecutive days for ZDF rats and 27 days for ZL rats. Because the glucose concentration of ZL rats showed no detectable fluctuations for over two weeks, the experimental period in them was one week shorter for the benefit of rats. Auricular margin was used as sham acupoint.

### ELISA

Concentration of plasma HbAlc was evaluated upon sacrifice at day 28 for ZL rats and day 35 for ZDF rats. ELISA kits were purchased from R&D System (Beijing, China) and analyzed by Huanya Biomedicine Technology Co. LTD (Beijing, China). The results were read using a microplate reader (Multiskan MK3, Thermo Scientific, Beijing, China) at wavelengths of 450 nm.

### Forced swimming test (FST)

The FST was modified based on the methods previously used in our study [18], [19] but omitted the pre-test session, which we found to be a confounding factor to the final results such that the pre-tested rats tended to stay still in the formal test and the immobility time was extremely prolonged to obscure the difference between animal groups. Briefly, a rat was placed in a clear plastic tank (45×35×60 cm) containing 30 cm of water (24±0.5°C) for 5 min. The total duration of non-swimming time within the 5-min session was recorded with a stopwatch as immobility score (in seconds) and compared among groups. A rat was judged to be non-swimming when no attempt was made to escape the tank and the rat was hunched forward (a floating position). Both ZL and ZDF rats underwent the same test procedure. Following FST session, the rat was removed from the water tank, dried with a towel, and returned to the home cage. All tests took place between 2 PM and 5 PM in a behavior testing room and were observed by the experimenter who blinded to the group assignment in order to minimize between-experimenter and between-session variations.

### Immunohistochemical staining

Rats were anesthetized with sodium pentobarbital and transcardially perfused with 200 ml of saline followed by 200–300 ml of cold 4% paraformaldehyde in 0.1 M phosphate buffer (PB). Brain sections from bregma −1.4 to −4 [20] were dissected, postfixed for 2 hr, and kept in 30% sucrose in 0.1 M PB until sank to the bottom. Tissues were then mounted in OCT compound and frozen on dry ice.

Brain (30 µm) sections were cut on a cryostat, mounted serially onto microscope slides, and stored at −80°C. Immunohistochemical staining was used to detect IR (mouse monoclonal, 1∶1000; Abcam, Cambridge, MA), NeuN (neuronal marker, rabbit monoclonal, 1∶1000; Abcam, Cambridge, MA), and GFAP (astrocyte marker, chicken polyclonal, 1∶1000; Abcam, Cambridge, MA). Sections were blocked with 1% goat serum in 0.3% Triton for 1 hr at room temperature and incubated overnight at 4°C with a primary antibody. For controls, a primary antibody was omitted. The sections were then incubated for 1 hr at room temperature with corresponding FITC- or CY3-conjugated secondary antibody (1∶200; Jackson ImmunoResearch, West Grove, PA). Four to six nonadjacent brain or spinal sections were randomly selected, analyzed using a LEXT OLS4000 3D Laser Measuring Microscope (Olympus), recorded using a digital camera, and processed using Adobe Photoshop.

### Western blot

The expression of IR in hypothalamus, liver, and skeletal muscle was tested by Western blot. Rats (n = 3 each group) were decapitated under anesthesia and samples were collected. The segments were homogenized in SDS sample buffer containing a mixture of proteinase inhibitors (Sigma). Protein samples were separated on SDS-PAGE gel (4–15% gradient gel; Bio-Rad, Hercules, CA) and transferred to polyvinylidene difluoride filters (Millipore, Bedford, MA). The filters were blocked with 3% milk and incubated overnight at 4°C with a primary antibody of IR (mouse monoclonal, 1∶4000; Abcam, Cambridge, MA) for 1 hr at room temperature with HRP-conjugated secondary antibody (1∶7000; Abcam, Cambridge, MA). The blots were visualized in ECL solution (NEN, Boston, MA) for 1 min and exposed onto hyperfilms (Amersham Biosciences) for 1–10 min. The blots were then incubated in a stripping buffer (67.5 mM Tris, pH 6.8, 2% SDS, and 0.7% β-mercaptoethanol) for 30 min at 50°C and reprobed with a polyclonal rabbit anti-GADPH antibody (1∶20,000; Abcam, Cambridge, MA) as loading control. Western analysis was made in triplicates. The density of specific bands was measured with a computer-assisted imaging analysis system and normalized against loading controls. Differences were compared using One-way ANOVA.

### Statistical analysis

By running GraphPad InStat version 3 for Windows, raw data of glucose concentration, immobility, and ELISA results were compared using repeated measure One-way ANOVA to detect differences among treatment groups, followed by Tukey-Kramer Multiple Comparisons Test to determine sources of differences. Data were presented as mean ± standard deviation. Differences were considered to be statistically significant at the level of α = 0.05. The correlation between HbAlc concentration and immobility time was acquired by linear regression.

## Results

### The development of hyperglycemia in naïve ZDF rats and the preventive effect of taVNS on the progression

**Figure 1:**
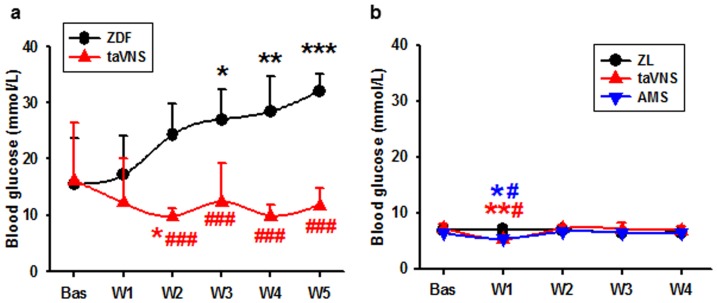
Blood glucose change in naïve and taVNS treated rats. Showing the development of hyperglycemia in ZDF rats with or without the taVNS treatment (a) and the effect of taVNS in ZL rats. ZDF, naïve ZDF rats; ZL, naïve ZL rats; taVNS, transcutaneous auricular vagus nerve stimulation; AMS, auricular margin stimulation; Bas, baseline; W1-5, week 1 to week 5 of the experiment. *, **, ***, P<0.05, 0.01, 0.001 *vs.* Bas; #, ###, P<0.05, 0.001 *vs.* naïve rats, respectively.

As determined by the weekly blood glucose concentration, the naïve ZDF rats (n = 6) developed hyperglycemia steadily with age, in contrast with a euglycemia in taVNS treated ZDF rats (Fig. 1a, n = 6). The results were also compared with that of ZL rats, naïve, and taVNS or AMS (auricular margin stimulation) treated rats (n = 4, each), in which no hyperglycemia developed (Fig. 1b).

### Effects of long term taVNS on depression-like behavior and plasma HbAlc concentration in rats

**Figure 2:**
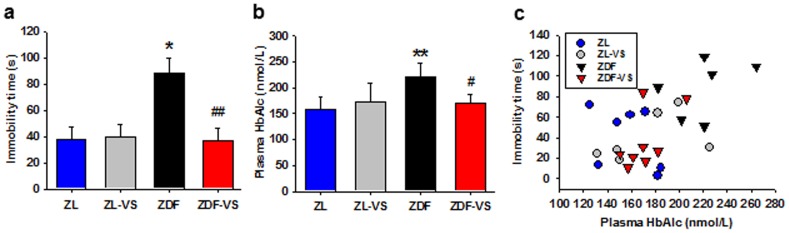
Correlation of depressive behavior and glucose metabolism dysfunction. Showing the immobility time of rats in FST (a) and the HbAlc concentration (b) on day 35 and their correlation in linear regression. ZL, naïve ZL rats; ZL-VS, taVNS in ZL rats; ZDF, naïve ZDF rats; ZDF-VS, taVNS in naïve ZDF rats. *, **, P<0.05, 0.01 *vs. ZL*; *#*, *##*, *P*<0.05, 0.01 *vs.* ZDF, respectively.

As determined by the FST on day 35, the naïve ZDF rats performed poorly in the tank with an immobility time of 87.83±28.03 (mean ± SD, n = 6). This immobility time is much longer than that of ZL rats and of taVNS treated ZDF rats (One-way ANOVA P = 0.006, Fig. 2a, Table 1). Simultaneously, the HbAlc concentration in taVNS treated rats is significantly lower than that in naïve ZDF rats (One-way ANOVA P = 0.0019, Fig. 2b, Table 2).

**Table 1:** Immobility time in FST on day 35.

Rat group	Naïve ZDF	ZDF-taVNS	Naïve ZL	ZL-taVNS
Mean	87.833	36.625	40	39.667
Standard deviation (SD)	28.032	28.096	29.878	23.304
Sample size (N)	6	8	7	6
Std. error of mean(SEM)	11.444	9.934	11.292	9.514
Lower 95% conf. limit	58.411	13.312	12.367	15.207
Upper 95% conf. limit	117.26	60.118	67.633	64.126
Tukey-Kramer multiple comparisons test P value *vs.* Naïve ZDF		<0.01	<0.05	<0.05

**Table 2:** HbAlc concentration on day 35.

Rat groups	Naïve ZDF	ZDF-taVNS	Naïve ZL	ZL-taVNS
Mean	219.57	170.94	157.68	172.91
Standard deviation (SD)	27.465	17.187	23.461	35.620
Sample size (N)	6	8	7	6
Std. error of mean(SEM)	11.213	6.076	8.867	14.542
Lower 95% conf. limit	190.74	156.57	135.98	135.53
Upper 95% conf. limit	248.40	185.31	179.38	210.30
Tukey-Kramer multiple comparisons test P value *vs.* Naïve ZDF		<0.05	<0.01	<0.05

The antidepressive and antidiabetic effects of taVNS were also evaluated in ZL rats. While it is antidepressive and antidiabetic in ZDF rats, the taVNS shows no effects in ZL rats, possibly due to normal swimming time and glucose metabolism condition in the latter (P>0.05, Fig. 2a, Table 1).

The correlation between the immobility time and the HbAlc concentration on day 35 is shown in Fig. 2c. As analyzed in linear regression, a strong positive correlation exists between the immobility time and the HbAlc concentration in ZDF rats (*y* = 153.244+0.685*x*), in contrast to ZL rats in which a weak negative correlation (*y* = 173.732−0.231*x*) is shown (Fig. 2C, Table 3). Alternatively, a higher HbAlc concentration can predicate a longer immobility time in ZDF rats (*y* = −108.276+0.87*x*), in contrast to that in ZL rats (*y* = 69.655−0.186*x*).

**Table 3:** Correlations between HbAlc level and immobility time in FST on day 35.

Rat groups	ZDF	ZL
Estimated HbAlc level from immobility time	*y* = 153.244+0.685*x*	*y* = 173.732−0.231*x*
Estimated immobility time from HbAlc level	*y* = −108.276+0.87*x*	*y* = 69.655−0.186*x*
Correlation	*R* = 0.757	*R* = −0.207
P value	*P* = 0.002	*P* = 0.497

### IR expression in naïve ZDF and the effect of taVNS on the expression of IR

**Figure 3:**
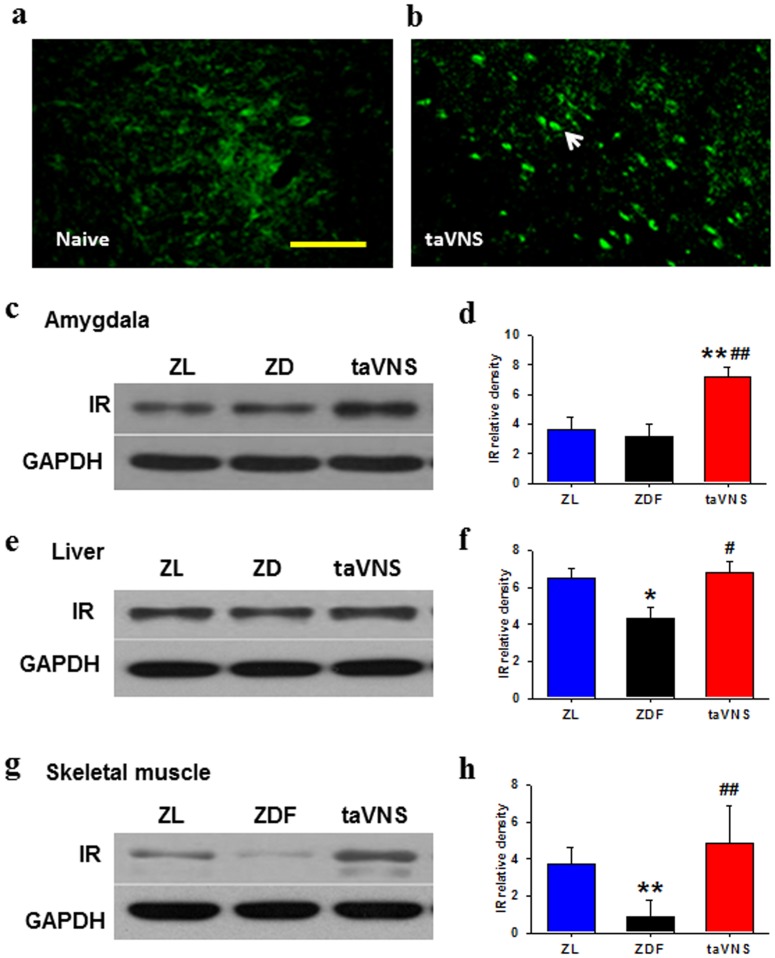
Expression of insulin receptor in various tissues of rats. (a, b) Immunofluorescent results showing the expression of insulin receptor **(IR)** in amygdala of naïve ZDF rats (a) and of ZDF rats treated for 5 consecutive weeks with daily half hour taVNS treatment (b). Arrow head, IR-immunopositive neurons. Bar, 100 µm. (c–h) Western blot results showing the expression of insulin receptor in hypothalamus (c, d), liver (e, f), and skeletal muscle (g, h) of naïve rats, or rats treated with taVNS for 34 consecutive days. ZL, naïve ZL rats; ZDF, naïve ZDF rats; taVNS, auricular vagus electronic stimulation. n = 3 each group, Western blot triplicated for each sample. *,** P<0.05, 0.01 *vs.* ZL; #,## P<0.05, 0.01 *vs.* ZDF.

As detected by immunostaining and by Western blots, the expression of IR in the hypothalamus, liver, and skeletal muscle is lower in naïve ZDF rats than that in ZL. However, in the same tissues of the taVNS treated ZDF rats, the IR expression was upregulated (Fig. 3).

**Figure 4:**
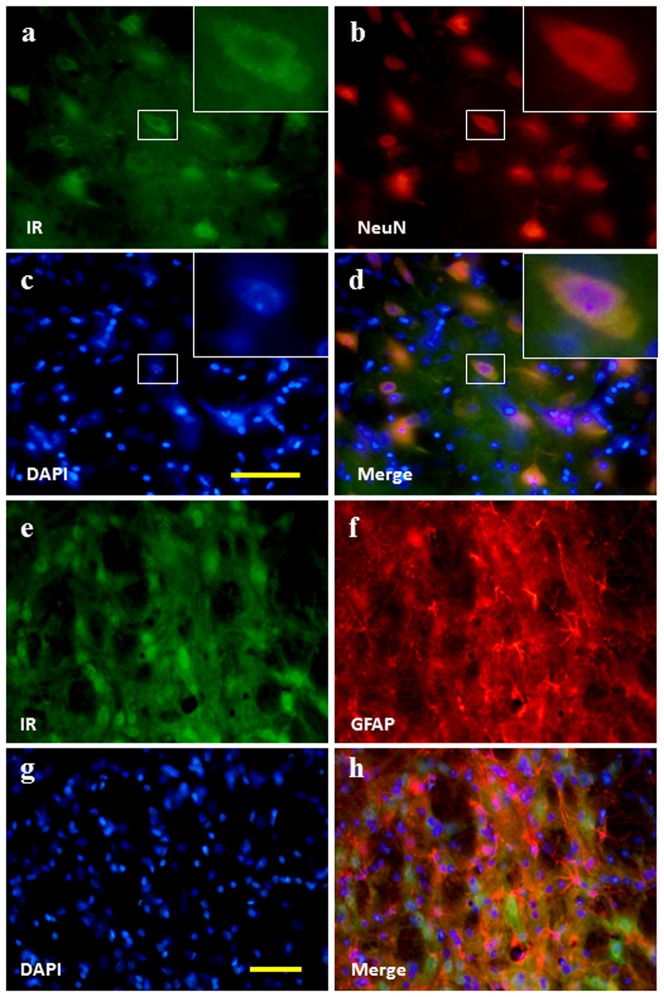
Neurochemical characteristics of insulin receptor positive cells in the brain. Double labelling immunofluorescence results showing that the IR is colocalized with NeuN (a–d) but not GFAP (e–h). Bar, 100 µm.

In double labelling immunofluorescence brain sections, the IR was colocalized with NeuN (Fig. 4a–d) but not GFAP (Fig. 4e–h), indicating that the IR-immunopositive cells in the brain is neuronal in characteristics. The IR expressed in the membrane of neurons.

## Discussion

In this study we report that the ZDF rats not only develop diabetes innately, they also develop depressive-like behaviors and that taVNS simultaneously prevent the progression of depressive behavior and hyperglycemia in ZDF rats.

A number of epidemiological studies have established a link between insulin resistance and the prevalence of depression. However, at this time it is not conclusive as to whether insulin resistance induces depression or the opposite. Although a study found that downregulation of hypothalamic insulin receptor expression elicits depressive-like behaviors in rats [21], others found that the occurrence of depression precede the onset of diabetes [22], and that insulin resistance can be reversed by antidepressant treatment [23]. Meanwhile, not only are depressive symptoms a risk factor for the development of T2D, they have also been shown to contribute to hyperglycemia, diabetic complications, functional disability and all-cause mortality among diabetic patients [4], [5]. Taken together, these reports indicate that the depression and insulin resistance may be bidirectional in cause.

Leptin is an adipocytokine or hormone secreted by white adipose tissue. Mediated through leptin receptor, leptin works together with insulin to decrease blood glucose level. Genetically, ZDF rats are leptin receptor deficient in and resistant to leptin, and therefor develop T2D with hyperglycemia and hyperinsulinemia. It is reported that hyperinsulinemia increases sympathetic nerve activity in obesity and diabetes [24] and that an increased sympathetic tone is closely related to depression [4]. From this point of view, we propose that depression in ZDF rats might be a result of hyperinsulinemia and glucose metabolism dysfunction in the CNS and that antidepressive effect of taVNS may be elicited by decreasing sympathetic tone or increasing parasympathetic activity; in turn, this antidepressive effect would also be helpful in reducing the insulin resistance. Indeed, the activity of the vagus nerve is negatively associated with risk factors such as stress and smoking, morbidity and mortality, and accordingly, taVNS which directly stimulates the afferent vagus branches at the auricular concha region, has been used in the therapeutic intervention in depression and epilepsy [23], [25], [26].

IR is necessary for the insulin to activate the intracellular signaling system both in the CNS and the peripheral cells. It is known that insulin performs unique functions within the CNS [27]. Produced nearly exclusively by the pancreas, insulin crosses the blood-brain barrier using a saturable transporter, affecting feeding and cognition through CNS mechanisms largely independent of glucose utilization. While peripheral insulin acts primarily as a metabolic regulatory hormone, CNS insulin has an array of effects on the brain that may more closely resemble the actions of the ancestral insulin molecule [28], [29]. Resistance to insulin action within the CNS may be in parallel or associated with peripheral insulin resistance, but it is also possible that variable insulin resistance syndromes exist so that resistance at one tissue may be independent from that of others. In this study, the expression of IR was low in various tissues including the hypothalamus, the liver, and the skeletal muscle of naïve ZDF rats and it was upregulated in taVNS treated rats. These results, while indicating that IR is involved in the pathogenesis mechanism of depression and T2D, also support the theory that taVNS is helpful in ameliorating both the depressive and the diabetic syndromes in these rats. It is very likely that, the upregulated expression of IR in the peripheral tissue such as liver and skeletal muscle is related to the amelioration of T2D, and in the CNS it is beneficial in the antidepressive effect of taVNS.

Although we demonstrate that taVNS is antidiabetic and antidepressive in ZDF rats, it has limited efficiency in ZL rats, a similar phenomenon as in melatonin treated experimental animals [30]. This may be due to a reduced parasympathetic activity in ZDF but not ZL rats. Further investigation would be focused on detection of the secretion change of hormones that are easily affected by the autonomic nervous system. One such hormone candidate is melatonin, which is related to both depression [19], [20], [31], [32] and T2D [33]–[35] with nonsufficient concentration in the circulating blood.

## Conclusions

Our results show that downregulated expression of IR and the compensatory hyperinsulinemia may be involved in the comorbidity between depression and T2D and that taVNS has a potential antidiabetic and antidepressive in ZDF rats.
